# Using Regional Climate Projections to Guide Grassland Community Restoration in the Face of Climate Change

**DOI:** 10.3389/fpls.2017.00730

**Published:** 2017-05-09

**Authors:** Kristin Kane, Diane M. Debinski, Chris Anderson, John D. Scasta, David M. Engle, James R. Miller

**Affiliations:** ^1^Department of Natural Resources and Environmental Science, University of Nevada RenoReno, NV, USA; ^2^Department of Ecology, Evolution and Organismal Biology, Iowa State UniversityAmes, IA, USA; ^3^Department of Agronomy, Iowa State UniversityAmes, IA, USA; ^4^Department of Ecosystem Science and Management, University of WyomingLaramie, WY, USA; ^5^Department of Natural Resource Ecology and Management, Oklahoma State UniversityStillwater, OK, USA; ^6^Department of Natural Resources and Environmental Sciences, University of IllinoisUrbana, IL, USA

**Keywords:** restoration, grasslands, Maxent, species distribution models, climate change

## Abstract

Grassland loss has been extensive worldwide, endangering the associated biodiversity and human well-being that are both dependent on these ecosystems. Ecologists have developed approaches to restore grassland communities and many have been successful, particularly where soils are rich, precipitation is abundant, and seeds of native plant species can be obtained. However, climate change adds a new filter needed in planning grassland restoration efforts. Potential responses of species to future climate conditions must also be considered in planning for long-term resilience. We demonstrate this methodology using a site-specific model and a maximum entropy approach to predict changes in habitat suitability for 33 grassland plant species in the tallgrass prairie region of the U.S. using the Intergovernmental Panel on Climate Change scenarios A1B and A2. The A1B scenario predicts an increase in temperature from 1.4 to 6.4°C, whereas the A2 scenario predicts temperature increases from 2 to 5.4°C and much greater CO_2_ emissions than the A1B scenario. Both scenarios predict these changes to occur by the year 2100. Model projections for 2040 under the A1B scenario predict that all but three modeled species will lose ~90% of their suitable habitat. Then by 2080, all species except for one will lose ~90% of their suitable habitat. Models run using the A2 scenario predict declines in habitat for just four species by 2040, but models predict that by 2080, habitat suitability will decline for all species. The A2 scenario appears based on our results to be the less severe climate change scenario for our species. Our results demonstrate that many common species, including grasses, forbs, and shrubs, are sensitive to climate change. Thus, grassland restoration alternatives should be evaluated based upon the long-term viability in the context of climate change projections and risk of plant species loss.

## Introduction

About one quarter of the Earth's terrestrial surface is covered by grasslands, and these are some of the most highly productive ecosystems in the world (Ramankutty and Foley, 1999; Bond, 2008). The inherent productivity of grassland soils has resulted in the conversion of large expanses of grassland to row crop agriculture (Hill and Olson, 2013; Wright and Wimberly, 2013). Woodland encroachment (Van Auken, 2000; Archer et al., 2001; Knapp et al., 2008) and urban expansion create additional threats (Kerns et al., 2016). The combination of such threats has relegated grassland ecosystem as one of the most endangered ecosystems in the U.S. (Noss et al., 1995; Samson et al., 1994). The ecological, sociological, and economic effects of grassland loss include threats to biodiversity (Lamarque et al., 2011; Ratajczak et al., 2012), losses in the suite of potential ways humans can interact with the landscape (Zheng et al., 2015), increased erosion, and a limited suite of economic land use options. While grasslands can be restored, particularly in places where soil is productive and native seeds can be collected and grown, the cost of restoration is often prohibitive. In some cases, removal of existing vegetation via herbicide or plowing is necessary prior to the reseeding. After establishment, management of grassland via fire and herbivores may be required to maintain the structure and composition of the native grassland (Fuhlendorf and Engle, 2001).

In the recent past, the goal in restoring an ecosystem was defined by baseline conditions (i.e., prior to human settlement) that generally included the species that had existed in the region historically (Hobbs and Norton, 1996). Once an ecosystem was restored, it was expected that, if the system was resilient, it could potentially stay in that state for decades or more (i.e., steady state). However, the threat of climate change has added a new complication. We can no longer simply replace the plant community that previously existed under historical conditions and assume that it will be successful. In the face of current knowledge about how ecosystems across the globe are changing with climate change (Field et al., 2014), ecologists now need an additional filter through which they must evaluate potential success. That is the filter of resilience to future climatic variation and future climatic change and anticipating species losses before they occur.

The Midwestern part of the United States is a place where grassland conversion has been extensive historically, even in the recent decade. Between 2006 and 2011, nearly 530,000 hectares (1.3 million acres) of grassland in the Western Corn Belt (WCB) were converted to cropland (Wright and Wimberly, 2013) for corn and soybean production. In response to this habitat loss and fragmentation, many grassland species are in decline (Jackson, 2001). As a result, there is also quite a bit of interest by natural resource managers in restoring and reconstructing grasslands.

The effects of climate change will potentially exacerbate the loss and fragmentation of grasslands. Global climate change models predict that future climate in the U.S. Midwest is expected to become highly variable (Wuebbles and Hayhoe, 2004). The pattern of precipitation over the next few decades is forecast to come in the form of large downpours, which translates into a higher likelihood of flooding, and increased intervals of drought between precipitation events (Meehl and Tebaldi, 2004). Increased temperatures and altered precipitation patterns are a threat to the biodiversity, the stability of grassland native plant communities (Thomas et al., 2004; Hampe and Petit, 2005) and the goods and services that grasslands provide (Adger et al., 2005; Stern, 2007). The inevitable consequence of changing species distributions and environmental alterations through climate and land use change, will be a higher proportion of “novel” or “emerging systems” (Harris et al., 2006; Root and Schneider, 2006; Hobbs et al., 2009), which will have significant implications for restoration and management practices.

Here, we use a case study of grassland restoration in the central part of the U.S. (Miller et al., 2012) to provide an example of how grassland restoration can be accomplished in a way that is resilient to climate change. We hypothesized that vegetation responses to forecasted climate change would be variable among photosynthetic pathways and plant functional group types and that by quantifying this variation recommendations for plant species selection and restoration can be guided by our modeling. We start with a suite of plant species known to exist in present grasslands and reconciled with field data. We then apply a fine scale climate model to this region, and evaluate the habitat suitability for each of these plant species under future conditions. This allows us to summarize which species will be most successful under future climate conditions and which species will not. This case study demonstrates the additional new planning step that will be needed to ensure successful long-term restoration of native grassland plant communities.

We used fine scale climate models and species distribution modeling to evaluate the future success of a suite plant species commonly associated with U.S. Midwestern grasslands. We used native perennial grasses such as [*Andropogon gerardii* (Big bluestem), *Schizachyrium scoparium* (Little bluestem), and *Sorghastrum nutans* (Indian grass)] as these three species are particularly characteristic of the tallgrass prairie of the Midwestern U.S. region. Species distribution modeling has been used for both conservation planning and theoretical research on ecological and evolutionary processes, and these analyses are primarily conducted at coarse geographic scales (Ferrier et al., 2002; Funk and Richardson, 2002; Loiselle et al., 2003; Rushton et al., 2004; Elith et al., 2006; Peterson, 2006; Kozak et al., 2008). In contrast, fine scale species distribution models have primarily been used for modeling distributions of a smaller number of either weedy or rare and endangered plant species (Collingham et al., 2000; Engler et al., 2004; Williams et al., 2009). These models establish relationships between occurrences of species and environmental conditions in the study area. A variety of species distribution modeling methods is available to predict potential habitat for a species (Guisan and Zimmermann, 2000; Kumar et al., 2006; Wisz et al., 2008). Each method is unique with regard to data requirements, statistical methods and overall ease of use (Guisan and Zimmermann, 2000). The prediction and mapping of potential habitat for threatened and endangered species can guide the monitoring and restoration of these declining native populations (Gaston, 1996).

We predicted habitat suitability responses to climate change for 33 species of grasses, forbs, shrubs, and woody species of the Midwestern tallgrass prairie ecosystem. Our objectives were to (1) develop models that estimate the relative suitability of habitat occupied currently by these species, (2) utilize these models to project change in the suitability of habitat, and (3) evaluate how these results might affect a manager's perspective on restoration within this ecosystem. We modeled responses at two time frames using climate change scenarios which differ dramatically in their predicted CO_2_ emissions. The larger goal of presenting this case study was to demonstrate the use of such downscaling techniques so that they could be applied in other grasslands worldwide.

## Methods

### Study region and species data

**Figure 1:**
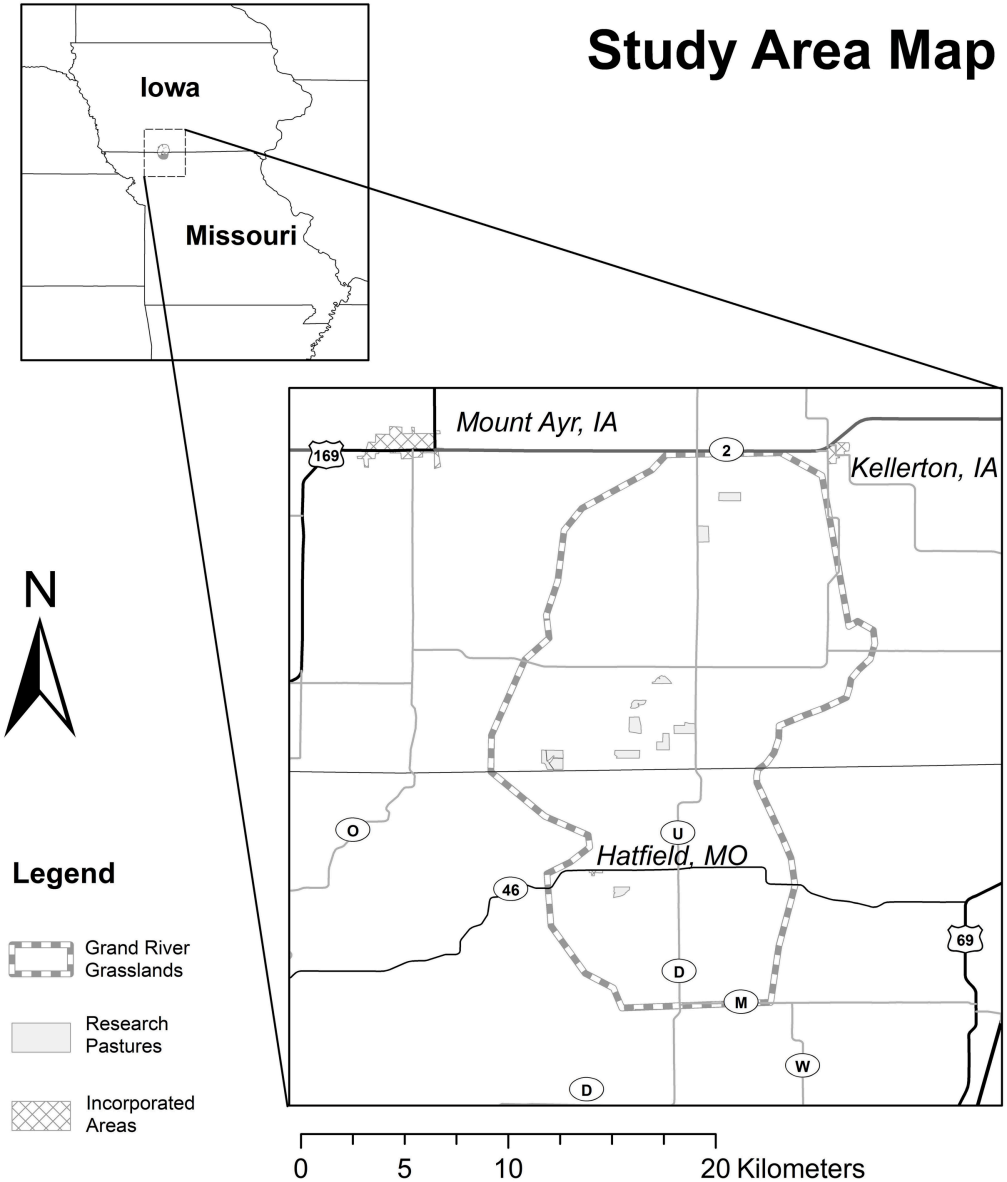
**Location of the experimental pasture plots in Grand River Grasslands study area in Ringgold County Iowa and Harrison County Missouri**.

The Grand River Grasslands (GRG) of Ringgold County, Iowa, and Harrison County, Missouri, is a 28,000 hectare conservation priority area comprised mostly of privately owned farms and ranches (Delaney et al., 2015; Figure 1). It has been identified as the best known opportunity to restore a functional tallgrass prairie system in the entire Central Tallgrass Prairie ecoregion (The Nature Conservancy, 2008). This Conservation Opportunity Area supports a diversity of grassland wildlife of conservation concern, including northern prairie skinks (*Plestiodon septentrionalis*), regal fritillary butterflies (*Speyeria idalia*), and grassland birds [(Greater Prairie-Chickens (*Tympanuchus cupido*), Henslow's Sparrows (*Ammodramus henslowii*), Dickcissels (*Spiza americana*), Bobolinks (*Dolichonyx oryzivorus*), Northern Harriers (*Circus cyaneus*)]. We obtained occurrence records of plant species in the GRG from Whittaker plot surveys conducted in 11 experimental pastures in Iowa and one in Missouri during May-August 2011 and 2012. Methodologies are described in McGranahan et al. (2012). We used a total of 33 plant species common to these pastures, including native and exotic warm-season and cool-season grasses, forbs, and woody species (Table S1). Occurrence records for each county in Iowa and one county in Missouri were obtained from The Biota of North America (BONAP), *North American Plant Atlas* (Kartesz, 2013).

### Predictor variables

We chose six predictor variables: temperature seasonality (represents seasonal variation in temperature), annual precipitation, precipitation of driest quarter, precipitation of the driest month, isothermality [mean diurnal range in temperature/(maximum temperature of warmest month—minimum temperature of coldest month)], and precipitation of wettest quarter. We chose these variables because they influence growth and survival patterns of plant species (Woodward, 1987; O'Donnell et al., 2012) and because these predictor variables were uncorrelated as Pearson correlations were <0.75 (Booth et al., 1994; Table 1). Current (1950–2000) and future climate time periods (2040's and 2080's) derived at 30 arc seconds (~1 km) resolution from the Worldclim dataset (Hijmans et al., 2005) were used for model simulation. Future climate scenarios were obtained from the Delta Method—a downscaling method based on thin plate spline spatial interpolation of anomalies (deltas) of original Global Climate Model (GCM) outputs (Hijmans et al., 2005). We compared species responses under two climate scenarios (A1B and A2). The A1B scenario predicts a linear increase in temperature from 1.4 to 6.4°C, and CO_2_ emissions remain stable at 15 gigatons of carbon from 2040 to 2080. Whereas, the A2 scenario predicts temperature increases from 2 to 5.4°C and CO_2_ emissions range from 16 Gigatons of carbon in 2040 to 23 Gigatons of carbon by 2080 (Field et al., 2014).

**Table 1:** **Selected environmental variables and their percent contribution to Maxent model for plant species in the Grand River Grasslands**.

**Bioclimatic variables**	**Percent contribution to model (%)**
Isothermality	34.2
Annual precipitation	27.1
Precipitation of the wettest quarter	20.6
Precipitation of the driest month	8.2
Temperature seasonality	5.7
Precipitation of the driest quarter	4.2

*The Percent Contribution column is an estimate of variable use relative to other variables in the model building process*.

### Modeling methods

We modeled species current and future distributions using the maximum entropy model Maxent version 3.3.3 k (Phillips et al., 2006; Phillips and Dudík, 2008). Maxent is considered a presence-background modeling technique because it does not require the use of absence data and it incorporates information on environmental variation across the study area (aka “background data”) during model development. Maxent has been compared to other presence-only methods and is regarded as the most reliable and robust (Elith et al., 2006; Hernandez et al., 2006; Wisz et al., 2008). This technique is also particularly effective when species sample sizes are small. Maxent uses occurrence data and environmental variables at those occurrence points to create distributional models. The environmental variables or features impose constraints on the unknown distribution so the mean of each feature is required to be within some error bounds of the empirical average over the presence sites. The constraints are relaxed using regularization parameters. Regularization prevents Maxent from “overfitting” occurrence points to environmental variables in order to avoid negative effects on predictive performance (Hastie et al., 2001). Models that are over-fit fail to predict independent evaluation data and transferability to another region or time period (Phillips and Dudík, 2008). Maxent approximates an unknown distribution using the known occurrences and background points and among all distributions satisfying the constraints, chooses the one of maximum entropy, or the most unconstrained one (Jaynes, 1957). In our study, the known distribution maps were developed for each species based on points/grid cell values across the 99 counties within the state of Iowa.

For each species, we modeled the current distribution and then projected it onto two sets of future time periods (2040 and 2080) and two emission scenarios (A1B and A2). We increased the level of regularization for each species to two (from a default setting of 1), as doing so alleviates overfitting when sample sizes are small and greatly increases model performance and model transferability to future climates (Dudík et al., 2007; Elith et al., 2010; Anderson and Gonzalez, 2011; Radosavljevic and Anderson, 2014). To ensure that our models did not extrapolate beyond the environmental envelop of the occurrence data used in model development, we ran the Maxent model using the default “clamping” option to generate mapping predictions which treat variables outside the training ranges as if they were at the end of their training range (Elith et al., 2011). The “donotextrapolate” option was also used as this sets predictions to zero whenever variables are outside of the training range. Lastly, we selected the logistic output format, which yields continuous values that indicate relative environmental suitability for the species (Phillips and Dudík, 2008). These values range from 0 (low probability of presence) to 1 (high probability of presence). Because we were modeling the future distributions within a relatively small geographic region (the Grand River Grasslands), we built our current distribution models using occurrence points from across all of Iowa to increase background data and ensure a broader representation of environmental conditions.

The predictive ability of all models was evaluated by using the area under the receiver-operator curve (AUC) which is a threshold-independent measure of predictive accuracy based only on the ranking of locations (Fielding and Bell, 1997). AUC is interpreted as the probability that a randomly chosen presence location is ranked higher than a randomly chosen background point (Merow et al., 2013). This approach corresponds to finding a model that identifies attributes of the species distribution and not artifacts of noise such as sampling bias. The AUC statistic measures the quality of a fitted model when calculated for the training data set, and it is a measure of the quality of prediction for novel environments. The AUC for our models ranged from good (0.7) to near perfect discrimination (≥0.9) (Table S1). If species had samples sizes of <25 records, models were tested and trained using the re-sampling K-fold cross-validation method, where the data are split into training data (to fit the model) and test data (to evaluate model predictions). Using this approach, the data are split into K independent subsets, where K is the number of replicates you specify, and one subset is left out while the model is fit to the other n-1 subsets. The subset withheld is used to test the model and calculate AUC (Elith et al., 2011). For species with samples sizes of >100, 25% of the training data were set aside for testing. If the number of species occurrence points was 10 or less, one data point was used for testing (Pearson et al., 2007).

## Results

**Figure 2:**
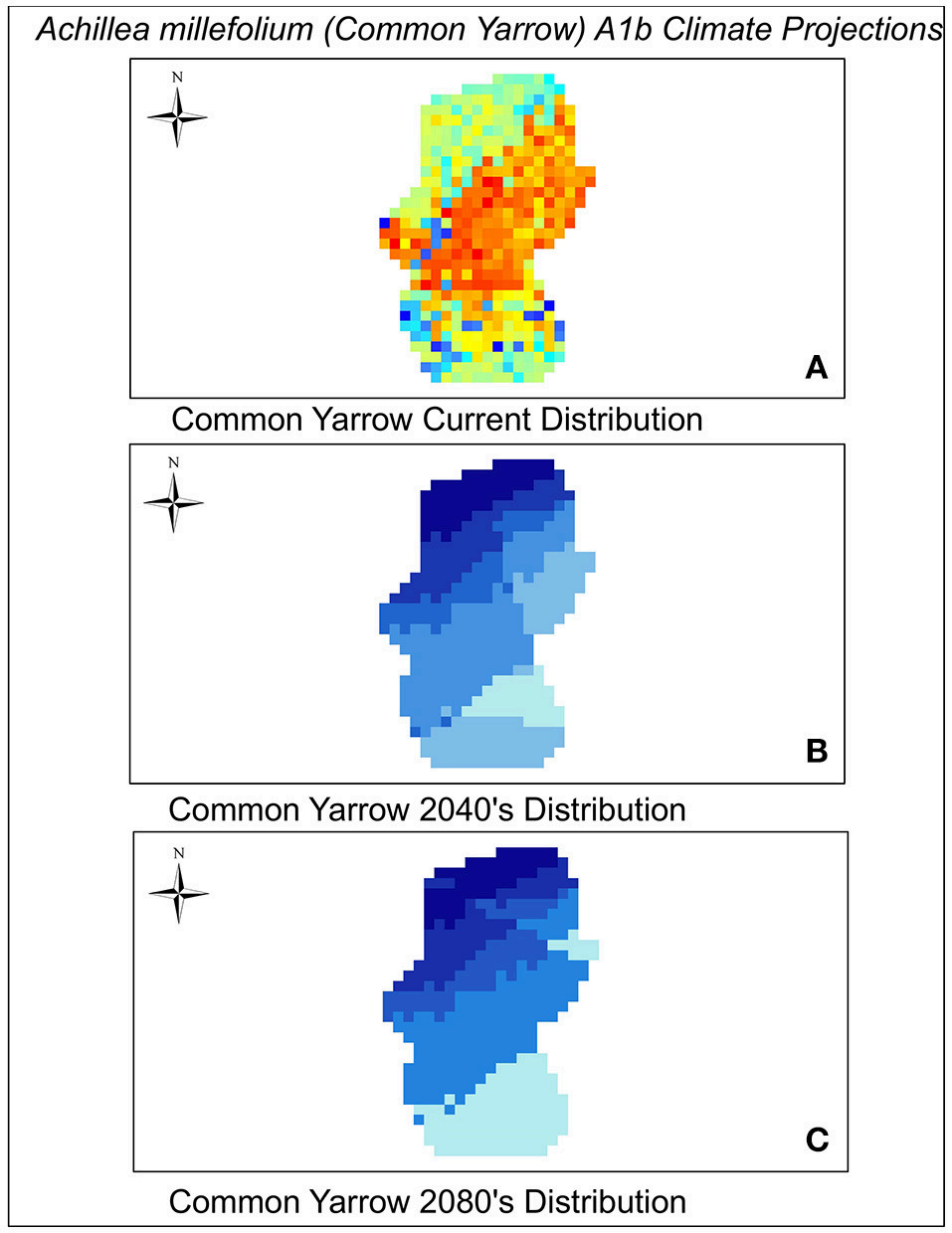
**Current (A)**, 2040 **(B)**, and 2080 **(C)** distribution of Common milkweed. A1 scenario predictions are coded blue (lowest suitability) to orange—red (highest suitability).

**Figure 3:**
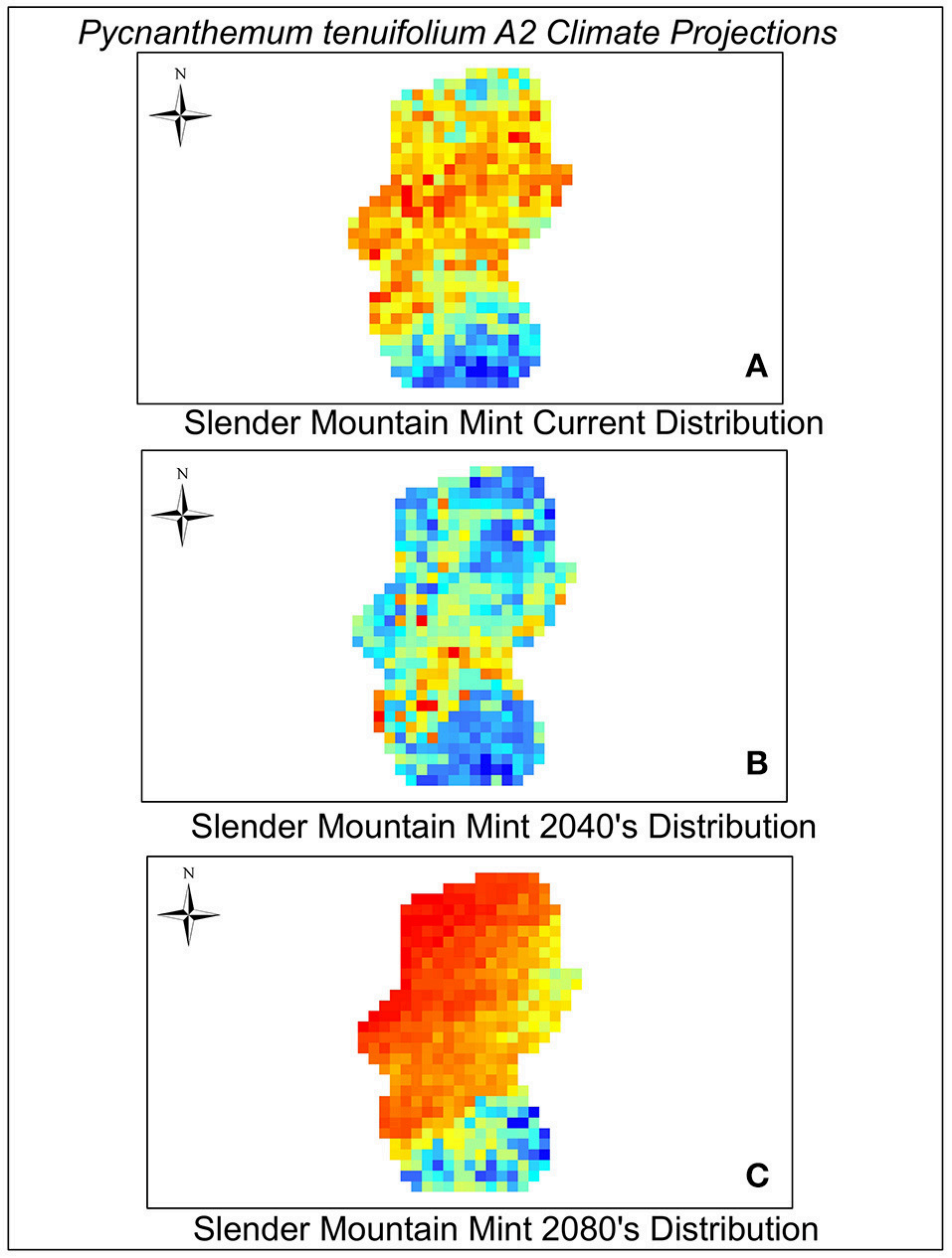
**Current (A)**, 2040 **(B)**, and 2080 **(C)** distribution of Slender Mountain Mint. A2 scenario predictions are coded blue (lowest suitability) to orange—red (highest suitability).

**Figure 4:**
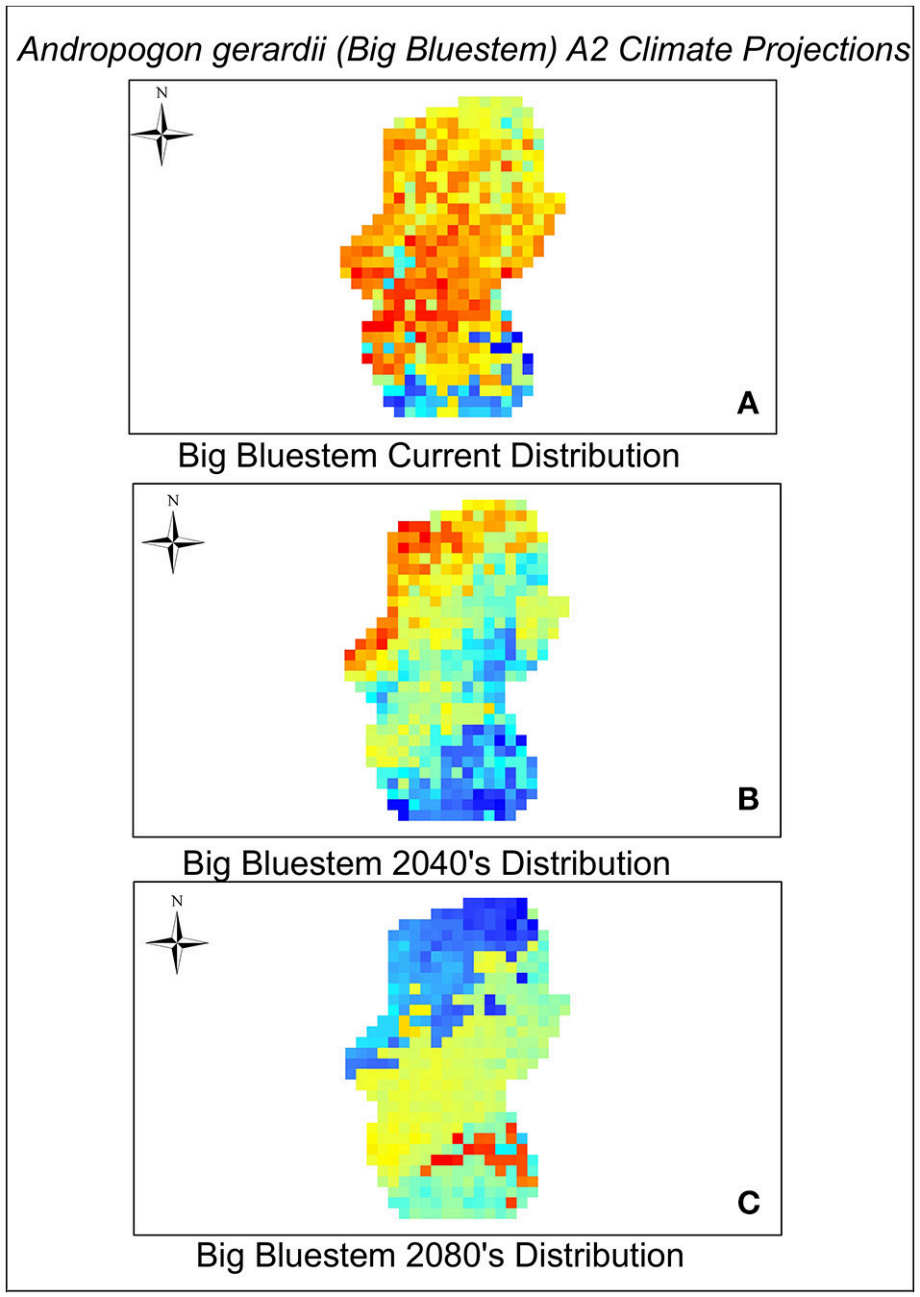
**Current (A)**, 2040 **(B)**, and 2080 **(C)** distribution of Big bluestem. A2 scenario predictions are coded blue (lowest suitability) to orange—red (highest suitability).

As with most climate change projection studies, model projections depend greatly on simulations of future climate, GHG emission levels, and species dispersal scenarios (Bakkenes et al., 2002). Our models were made using the IPCC global emissions climate scenarios A1B and A2. The A1B scenario predicts a temperature increase of as much as 5°C between the years 2040 and 2080 whereas the A2 scenario predicts a 3.4°C temperature increase but larger changes in CO_2_ emissions (increases by much as 7 gigatons of carbon/year between 2040 and 2080). In addition, the variable isothermality made the greatest contribution to our Maxent model. Isothermality quantifies the range between the day-to-night temperature oscillation and summer to winter oscillation. We found that the values of isothermaility in the A1B scenario decreased by a factor of 84% in both the 2040 and 2080 climate scenarios in comparison to the current scenario. This was not observed in the A2 scenario. Annual precipitation increased in both scenarios from the year 2012. Annual precipitation in the A1b scenario increased by 70 mm in 2040 and by 79 mm in 2080. Under the A2 scenario precipitation increased by 92 mm of precipitation per year (2040 and 2080). The A1B scenario was the most severe, predicting very low values of habitat suitability and low variation in these values across species in both time periods (Table 2). The A2 scenario predicted higher habitat suitability scores and more variation in those scores among the species for both time periods. However, both models predict the majority of species will experience declines in habitat by 2040. Projections to 2080 show that suitable habitat may only be available for a small subset (~12%) of the 33 species considered here (Table 2). We have provided a view of habitat suitability for two examples of common forbs, *Achillea millefolium* (yarrow) and *Pycnanthemum tenuifolium* (slender mountain mint) and one example of a common grass, *S. scoparium* (big bluestem) found in Midwestern prairies and grasslands (Figures 2–4). The full set of results for all 33 species can be viewed in Figures S1–S4.

**Table 2:** **Plant species modeled and relative habitat suitability values under the A2 and A1B scenario**.

			**A2**		**A1B**	
**Species**		**2012**	**2040**	**2080**	**2040**	**2080**
*Achillea millefolium*	(common yarrow)	0.90	0.75	0.16	0.01	0
*Ambrosia artimisiifolia*	(common ragweed)	0.90	0.01	0	0.01	0
*Andropogon gerardii*	(big bluestem)	0.86	0.63	0.10	0	0
*Antennaria neglecta*	(pussy toes)	0.86	0	0	0	0
*Aster ericoides*	(aster heath)	0.96	0.16	0	0	0
*Asclepias syriaca*	(common milkweed)	0.84	0.84	0.24	0	0
*Bromus inermis*	(smooth brome)	0.90	0	0	0.01	0
*Daucus carota*	(wild carrot)	0.90	0.71	0.13	0.01	0
*Dicanthelium spp*.	(panic grass)	0.90	0.79	0.21	0.78	0.21
*Dactylis glomerata*	(orchard grass)	0.90	0.78	0.24	0.10	0.10
*Erigeron strigosus*	(daisy fleabane)	0.90	0	0	0	0
*Schedonorus arundinacea*	(tall fescue)	0.90	0	0	0	0
*Fragaria virginiana*	(wild strawberry)	0.90	0	0	0.01	0
*Lotus corniculatus*	(birdsfoot trefoil)	0.90	0.75	0.11	0	0
*Maclura pomifera*	(osage orange)	0.90	0	0	0	0
*Monarda fistulosa*	(wild bergamot)	0.90	0	0	0.01	0
*Panicum virgatum*	(switchgrass)	0.90	0	0	0.01	0
*Phleum pratense*	(timothy)	0.88	0	0	0	0
*Plantago rugelii*	(plantain blackseed)	0.87	0	0	0	0
*Poa pratensis*	(Kentucky bluegrass)	0.90	0	0	0.01	0
*Potentilla simplex*	(common cinquefoil)	0.90	0.20	0.04	0	0
*Pycanthemum tenuifolium*	(slender mountain mint)	0.90	0.44	0.90	0	0
*Ratibida pinnata*	(gray headed coneflower)	0.84	0	0	0	0
*Schizachyrium scoparium*	(little bluestem)	0.80	0.80	0.10	0	0
*Sorghastrum nutans*	(Indian grass)	0.80	0.80	0.20	0.80	0.20
*Sporobolus clandestinus*	(rough dropseed)	0.80	0.80	0.20	0	0
*Symphoricarpos orbiculatus*	(buckbrush)	0.90	0.10	0.80	0	0
*Toxicodendron radicans*	(poison ivy)	0.90	0.10	0.50	0	0
*Trifolium pretense*	(red clover)	0.90	0	0	0	0
*Trifolium repens*	(white clover)	0.90	0	0	0.91	0
*Vernonia baldwinii*	(Baldwin's ironweed)	0.90	0.20	0.90	0.20	0.90
*Viola pedata*	(birdsfoot violet)	0.90	0	0	0.01	0
*Viola pedataifida*	(prairie violet)	0.90	0	0	0.01	0

*Relative environmental suitability for the species ranges from 0 (lowest suitability score) to 1 (highest suitability score)*.

Models based on the A1B scenario show that by both 2040 and 2080 only 10% or less of currently available habitat will be suitable in the Grand River Grassland for many plant species (Table 2, Table S1). The few species with relatively high habitat suitability in 2040 such as *Dicanthelium* spp. (panic grass), *S. nutans* (Indian grass), and *Trifolium repens* (white clover) are predicted to undergo a substantial decrease to their habitat suitability by 2080. The only species that shows high habitat suitability from in 2080 is *Vernonia baldwinii* (Baldwin's ironweed).

Models run using the A2 scenario showed that only four plant species [*P. tenuifolium* (Slender mountain mint), *Symphoricarpos orbiculatus* (Buckbrush), *Toxicodendron radicans* (Poison ivy), and *V. baldwinii* (Baldwin's ironweed)] are expected to experience declines in habitat suitability by 2040 (Table 2, see Figure 3 as an example and Figure S2), but then show increases in habitat suitability between 2040 and 2080. Given that species have to pass through low habitat suitability conditions in 2040 to get to higher 2080 suitability conditions it is possible that they may not have the chance to recover. Ten species (four warm-season grasses, two cool-season grasses, and four forbs) show no change in habitat suitability by 2040 but suitability will decline substantially by 2080 (Table 2, Figure S3). Of particular note relative to Midwestern grassland restorations is that perennial grasses [*A. gerardii* (Big bluestem), *S. scoparium* (Little bluestem), *S. nutans* (Indian grass), and *Sporobolus clandestinus* (Rough dropseed)] show a much more dramatic reduction in habitat suitability between the two time periods. And similarly, relative to current conservation issues related to monarch butterfly (*Danaus plexippus*) restoration, even common milkweed, *Asclepias syriaca*, suffers low habitat suitability by 2080. For all other species, suitable habitat begins to decline by 2040 and continues to decline by 2080 (Table 2, Figure S4).

## Discussion

Our case study demonstrates that even the most common plants in the Midwestern region of the U.S. are vulnerable to climate change and that we have a rapidly shrinking time window to understand how to conserve and restore these critical ecosystems. The decline in suitability for native perennial grasses such as [*A. gerardii* (Big bluestem), *S. scoparium* (Little bluestem), and *S. nutans* (Indian grass)] under both scenarios is particularly troubling. If significant changes are projected for even the most common species, the implications of climate change mean that there could be state transitions in the plant and animal communities associated with Midwestern grasslands in the coming decades (Briske et al., 2005).

The A1b and A2 scenarios delivered different results. We believe these differences are driven by a decrease in the diurnal temperature range (DTR) of our study region. In the current scenario, the isothermality values indicate that the DTR and the annual temperature range were about even. In the 2040 A2 scenario the isothermality values plunged, indicating that the DTR decreased below the annual temperature range. These changes in DTR can directly affect photosynthesis, respiration, growth and tissue restoration in plants (Hughes, 2000).

Because these projected changes have ramifications for agriculture, conservation, and society in general, our results imply that it is imperative that future land management in this region incorporate climate variability as a driver of plant communities (Scasta and Rector, 2014). Current national and regional frameworks for land management have very few ecological site descriptions (ESD's) and state-and-transition models that include climate change (Twidwell et al., 2013). It also begs the question of how resilient we should expect grasslands to be in other parts of the world where seed sources, precipitation, and soil quality are more limiting.

The reduction of some of the plant species in this Midwestern U.S. example could have important implications for pollinator species and other wildlife. For example, three common species, *S. orbiculatus* (Buckbrush), and *V. baldwinii* (Baldwin's ironweed) and *P. tenuifolium* (Slender mountain mint) are expected to decline in habitat suitability under 2040 projected models. Both slender mountain mint and Baldwin's ironweed are important nectar sources for pollinators (Kopper et al., 2001), so a decline in the abundance of these species could cascade across other taxa such as butterflies and bees. Similarly, Buckbrush is one of the most common shrubs in these prairies, providing nesting habitat for some grassland birds and browse for deer (Soper et al., 1993; Holechek, 2001; Coppedge, 2010). Declines in Buckbrush could thus have important effects on birds and wildlife. The predicted decline in Buckbrush is also surprising because it is resilient to regular disturbance such as fire, even during periods of extreme drought like 2012 (Scasta et al., 2014). We should note that in some of these cases species showed declines in habitat suitability in 2040 followed by increases in habitat suitability in 2080. It is essential to note that in a real world scenario, if populations decline in 2040, they may not be resilient enough to increase by 2080.

Given the results that we obtained, some specific recommendations for this study region can be made. Dominant grasses in the tallgrass prairie region include [*A. gerardii* (Big bluestem), *S. scoparium* (Little bluestem), and *S. nutans* (Indian grass)]. Of these three species, *S. nutans* is the species that has the highest habitat suitability across all of the scenarios in the future, so it may be wise for managers to consider increasing the proportion of this species in future plantings. Dominant forbs (flowering plants) native to tallgrass prairies that we evaluated here include species such as *A. millefolium* (common yarrow), *Monarda fistulosa* (wild bergamot), *Ratibida pinnata* (gray headed coneflower), and *Potentilla simplex* (common cinquefoil). Of these flowering plants, *A. millefolium* and *P. simplex* are the two species that have the highest habitat suitability in the future and might similarly be considered as good candidates to increase in future seed mixtures. Using this example as a model, managers of other grasslands could use a similar approach to revise the prescriptions used for restoration seed mixes.

Our analysis was not extensive enough to make broad generalizations among plant photosynthetic pathways given the limited number of species and C3 and C4 graminoids that both exhibit similar suitability responses across models [i.e., *Dichanthelium* species (panic grass) and *S. nutans* (Indian grass)]. Similarly, broad generalizations regarding functional group responses are difficult because different species within a functional group display both positive and negative suitability responses across models. No clear trend for the responses of invasive species was evident either. However, we can provide some insights based on the species we did examine. We included two woody species *Maclura pomifera* (osage orange) and *S. orbiculatus* (Buckbrush). Both species show declines, but the decline of *M. pomifera*, a tree species, is more dramatic than that for *S. orbiculatus*, a shrub species, particularly under Model A2. Similarly, we only examined one invasive species *Schedonorus arundinacea* (tall fescue), which is a grass frequently planted to pastures in the region. Habitat suitability for *S. arundinacea* drops to zero in both models and both time periods. A reduction in the cover of an invasive grass species may be considered a potential benefit by managers. Finally, *T. radicans* (poison ivy) is not an exotic species, but it is a vine-forming species that can cause an itchy rash after skin contact. The predicted decline in habitat suitability, particularly under Scenario A1B, would be considered a potential benefit by many land managers.

In addition to thinking about seed mixes for restoration and the potential survival of particular plant species, adjustments to grassland management practices may be in order. For example, if conditions are getting hotter and drier, current fire, and grazing management practices may have different effects on the plant communities than they have had in the past. Similarly, changing precipitation regimes may influence biomass production. As such, it may be appropriate to lengthen the fire-return interval, change the timing of burning, and/or modify stocking rates on grazed grasslands (e.g., Holechek et al., 1995).

In further interpreting these results, it is essential that we take into consideration the model assumptions. The bioclimatic models implemented in this study make a number of simplifying assumptions that may bias the projections (Pearson and Dawson, 2003; Guisan and Thuiller, 2005). There are several factors that would exacerbate the projected impacts of climate change, which our models ignore. These include specialization to restricted soil types (Harrison et al., 2006), the spread of invasive species, which could take over otherwise potentially appropriate habitat (Seabloom et al., 2006), local adaptation of populations within species, and genetic constraints on evolutionary response to climate change (Etterson and Shaw, 2001). On the other hand, resilience of established plants and seed banks (Chapin and Starfield, 1997), differing population responses at range margins (Hampe and Petit, 2005), and adaptive evolutionary responses might mitigate the influence of climate change. Also, we need to take into consideration the effect of grain size on our models, as there simply are not many types of climate data available for a study area of this size. However, our results point out the value of using regional approaches to understanding climate change effects. Finally, parsing out the regulating effects of climate and land-use change is difficult, particularly in the context of the removal of fire from the landscape and such effects on woody vs. non-woody vegetation (Archer et al., 1995). Both climate and land-use change will affect species distributions (Halpin, 1997), but it is not clear how the cumulative effects of these dual threats will manifest themselves in terms of future species responses.

Nonetheless, the outcomes from our modeling example provide a sobering perspective relative to long term sustainability of grasslands in the central portion of North America. Ecologists and managers will indeed need to add this new filter of regional habitat suitability under climate change as they manage for the future. Because large-scale grassland restorations are often costly endeavors, both in terms of human time and financial investments, ecologists will need to know whether they can expect restorations to be successful under future climatic conditions. We have thus developed the following recommendations:

- To ensure resilience in the face of climate change, take a proactive perspective to restoring grasslands, thinking about which species are expected to do well in the future and what geographic regions will be expected to provide suitable growing conditions. It would also be prudent to take a larger geographic perspective when thinking about restoration of particular communities. Model results could be used to triage restoration efforts toward both species and locations that are most likely to provide future habitat.
- Adjustments to management practices may be in order. Fire and grazing management practices may have different effects on the plant communities than they have had in the past and changing climatic patterns may influence ecosystem responses to disturbance. The effects of such changes should be considered when evaluating how grasslands respond to management.

In essence, ecologists and grassland managers will have to adjust our thinking to this “new normal” using our prior understanding of how communities function, but incorporating these new filters that describe how grasslands may function in the future.

## Author contributions

DD and CA conceived the research. CA provided guidance on downscaling of the climate data. KK designed and implemented the methods and analyzed the data. KK and DD wrote the manuscript. DD oversaw the research, and reviewed the manuscript; JS, JM, and DE provided data and edited the manuscript.

## Funding

The project described in this publication was supported by Grant No. [G12AC20504] from the United States Geological Survey to CA and DD. Its contents are solely the responsibility of the authors and do not necessarily represent the views of the North Central CSC or the USGS. This manuscript is submitted for publication with the understanding that the United States Government is authorized to reproduce and distribute reprints for Governmental purposes.

### Conflict of interest statement

The authors declare that the research was conducted in the absence of any commercial or financial relationships that could be construed as a potential conflict of interest.

## Supplementary material

Table S1 **List of 33 plant species modeled and the number of training samples used to build each model**. The test AUC is a measure of model performance at ±1 SD.

Figure S1–S4 **The full set of results for all 33 modeled plant species using the A1B and A2 climate scenarios**.
