# Supplementary material for: Using Regional Climate Projections to Guide Grassland Community Restoration in the Face of Climate Change
Source: Front Plant Sci. 2017 May 9;8:730. doi: 10.3389/fpls.2017.00730 (PMC5422548; doi:10.3389/fpls.2017.00730)
Supplement: Table S1 — List of 33 plant species modeled and the number of training samples used to build each model. The test AUC is a measure of model performance at ±1 SD. [file Table1.DOCX]

Table S1 List of 33 plant species modeled and the number of training samples used to build each model. The test AUC is a measure of model performance at +-1 SD.

|  |  | Training  samples | | Training  AUC | Test  AUC | Functional  Group | Origin |
| --- | --- | --- | --- | --- | --- | --- | --- |
|  | *Achillea millefolium*  (common yarrow) | | 135 | 0.8966 | 0.7435 | Forb | Native |
|  | *Ambrosia artimisiifolia*  (common ragweed) | | 119 | 0.8844 | 0.756 | Forb | Native |
|  | *Andropogon gerardii*  (big bluestem) | | 106 | 0.8894 | 0.7107 | C4 Grass | Native |
|  | *Antennaria neglecta*  (pussy toes) | | 12 | 0.7897 | 0.6868 | Forb | Native |
|  | *Asclepias syriaca*  (common milkweed) | | 130 | 0.9083 | 0.71 | Forb | Native |
|  | *Aster ericoides*  (aster heath) | | 34 | 0.8967 | 0.7432 | Forb | Native |
|  | *Bromus inermis*  (smooth brome) | | 23 | 0.8817 | 0.7682 | C3 Grass | Exotic |
|  | *Daucus carota*  (wild carrot) | | 123 | 0.8744 | 0.9352 | Forb | Exotic |
|  | *Dactylis glomerata*  (orchard grass) | | 119 | 0.9228 | 0.8195 | C3 Grass | Exotic |
|  | *Dicanthelium spp.*  (panic grasses) | | 116 | 0.8966 | 0.7433 | C3 Grass | Native |
|  | *Erigeron strigosus*  (daisy fleabane) | | 18 | 0.8966 | 0.7436 | Forb | Native |
|  | *Schedonorus* *arundinacea*  (tall fescue) | | 22 | 0.8945 | 0.7651 | C3 Grass | Exotic |
|  | *Fragaria virginiana*  (wild strawberry) | | 12 | 0.812 | 0.7464 | Forb | Native |
|  | *Lotus corniculatus*  (birdsfoot trefoil) | | 120 | 0.9003 | 0.7968 | Forb | Exotic |
|  | *Maclura pornifera*  (osage orange) | | 18 | 0.9262 | 0.8446 | Tree | Native |
|  | *Monarda fistulosa*  (wild bergamot) | | 21 | 0.8895 | 0.7483 | Forb | Native |
|  | *Panicum virgatum*  (switchgrass) | | 120 | 0.7351 | 0.5881 | C4 Grass | Native |
|  | *Phleum pretense*  (timothy) | | 19 | 0.9163 | 0.8039 | C3 Grass | Exotic |
|  | *Plantago rugelii*  (plantain blackseed) | | 17 | 0.8342 | 0.7688 | Forb | Native |
|  | *Poa pratensis*  (Kentucky bluegrass) | | 133 | 0.8843 | 0.756 | C3 Grass | Exotic |
|  | *Potentilla simplex*  (cinquefoil, common) | | 16 | 0.923 | 0.8389 | Forb | Native |
|  | *Pycanthemum tenuifolium*  (slender, mountain mint) | | 19 | 0.894 | 0.7543 | Forb | Native |
|  | *Ratibida pinnata*  (coneflower gray headed) | | 13 | 0.8914 | 0.7017 | Forb | Native |
|  | *Schizachyrium scoparium*  (little bluestem) | | 125 | 0.8888 | 0.7028 | C4 Grass | Native |
|  | *Sorghastrum nutans*  (Indian grass) | | 127 | 0.8874 | 0.6879 | C4 Grass | Native |
|  | *Sporobolus clandestinus*  (rough dropseed) | | 119 | 0.8884 | 0.754 | C4 Grass | Native |
|  | *Symphoricarpos orbiculatus*  (buckbrush) | | 19 | 0.9 | 0.7365 | Shrub | Native |
|  | *Toxicodendron radicans*  (poison ivy) | | 11 | 0.7923 | 0.7052 | Sub Shrub | Native |
|  | *Trifolium pretense*  (red clover) | | 18 | 0.9031 | 0.7685 | Forb | Exotic |
|  | *Trifolium repens*  (white clover) | | 1 | 0.9083 | 0.8071 | Forb | Exotic |
|  | *Vernonia baldwinii*  (Baldwin’s ironweed) | | 19 | 0.8941 | 0.7435 | Forb | Native |
|  | *Viola pedata*  (birdsfoot violet) | | 18 | 0.9901 | 0.8792 | Forb | Native |
|  | *Viola pedataifida*  (prairie violet) | | 1 | 0.9901 | 0.8792 | Forb | Native |
