# Supplementary figures and images for: Using Regional Climate Projections to Guide Grassland Community Restoration in the Face of Climate Change
Source: Front Plant Sci. 2017 May 9;8:730. doi: 10.3389/fpls.2017.00730 (PMC5422548; doi:10.3389/fpls.2017.00730)

**Supplement A**

A1b Scenario:


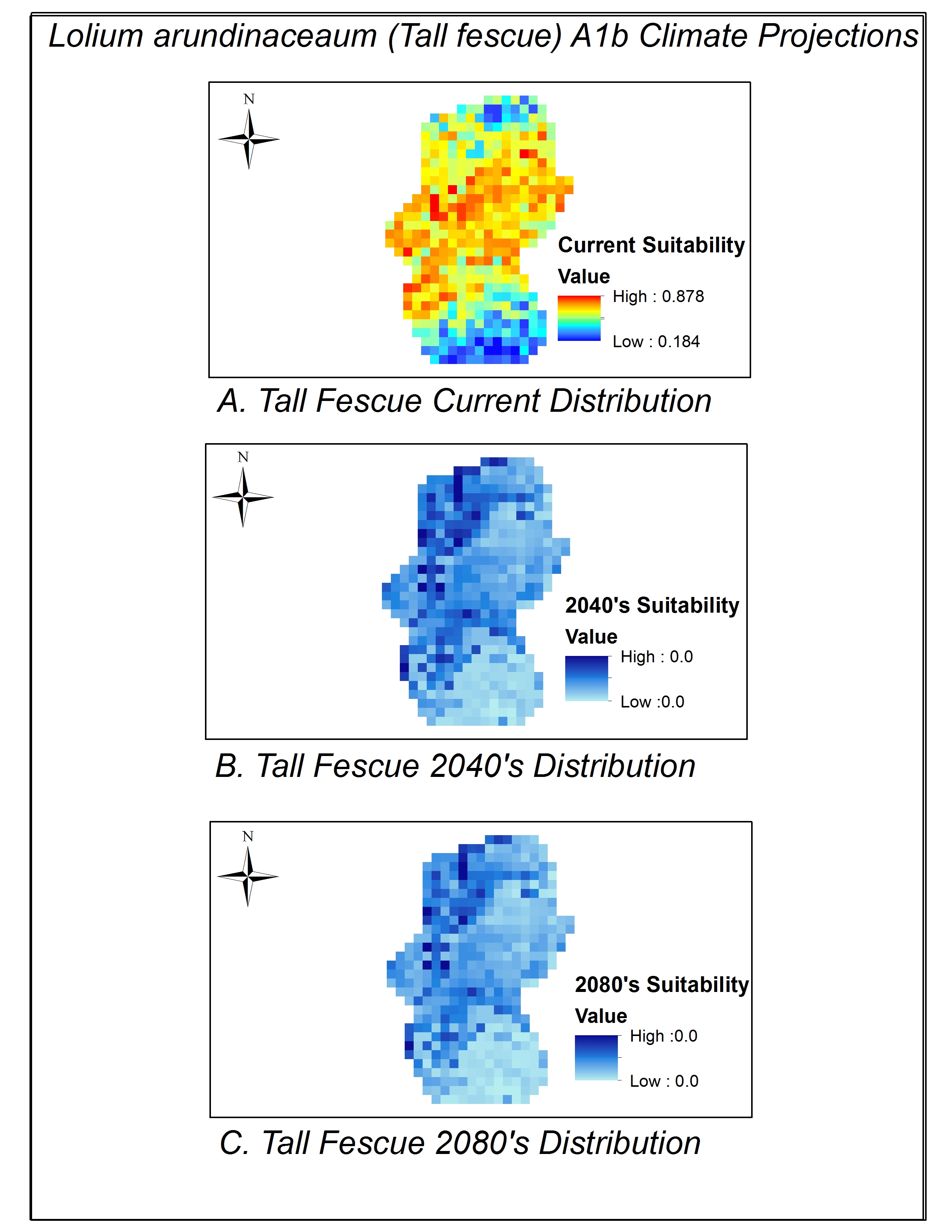


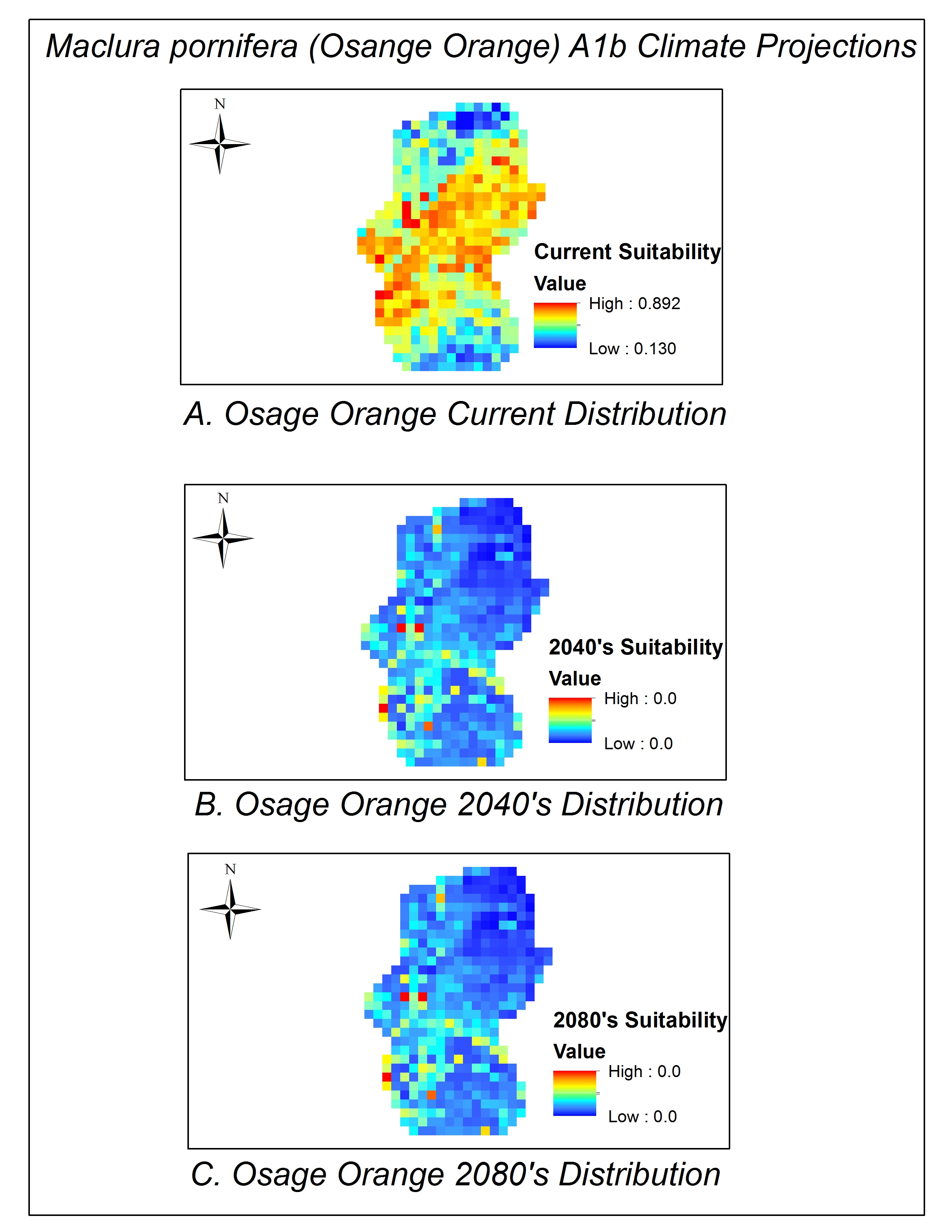


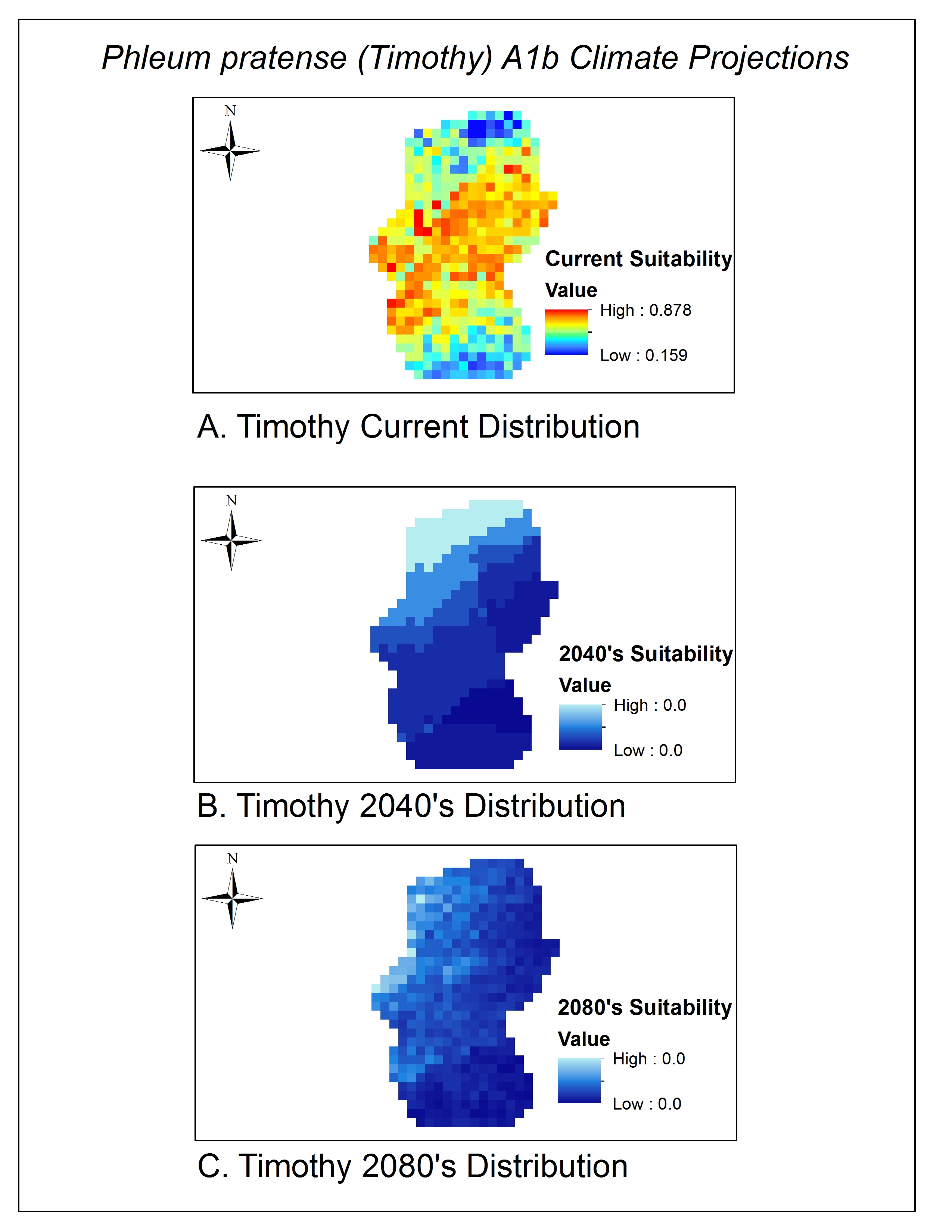


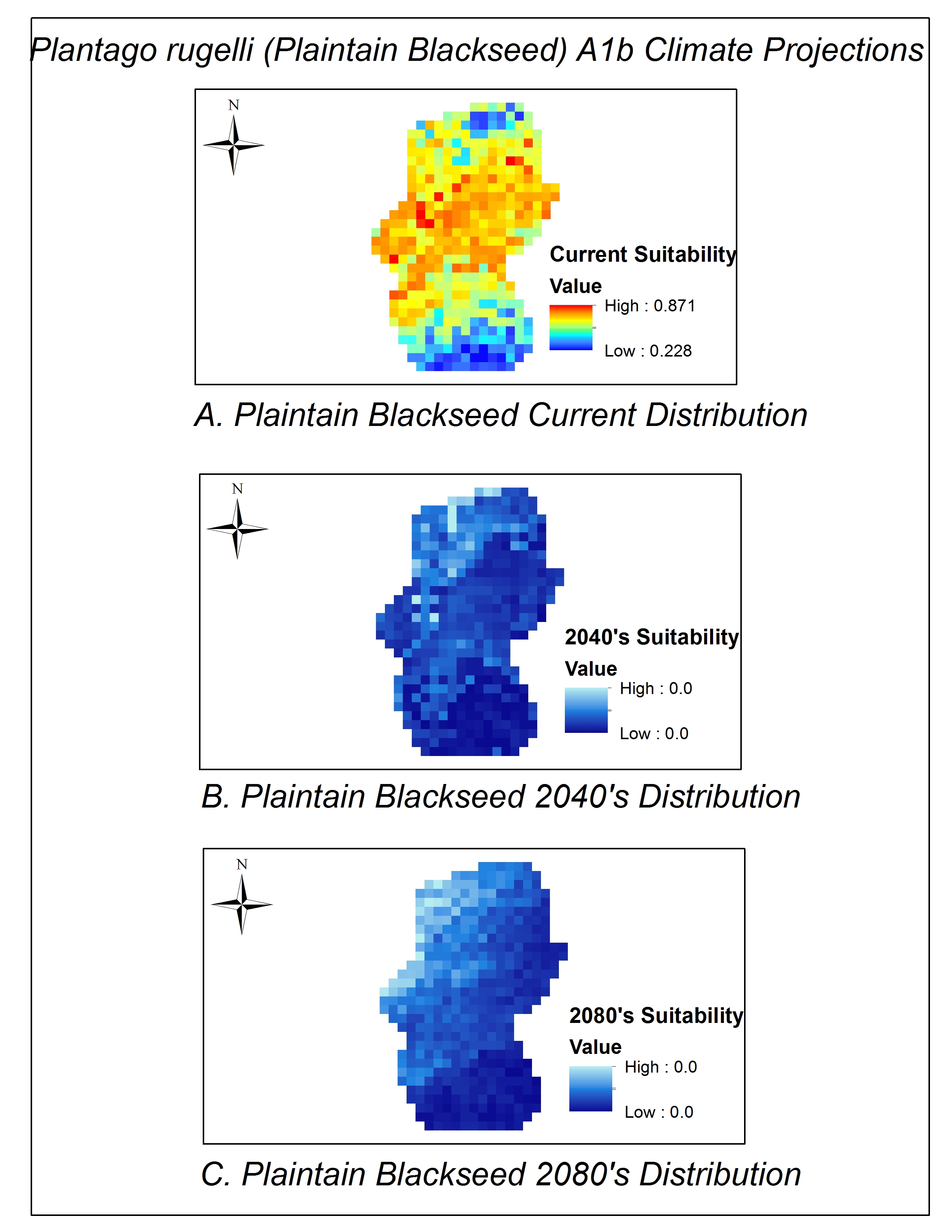


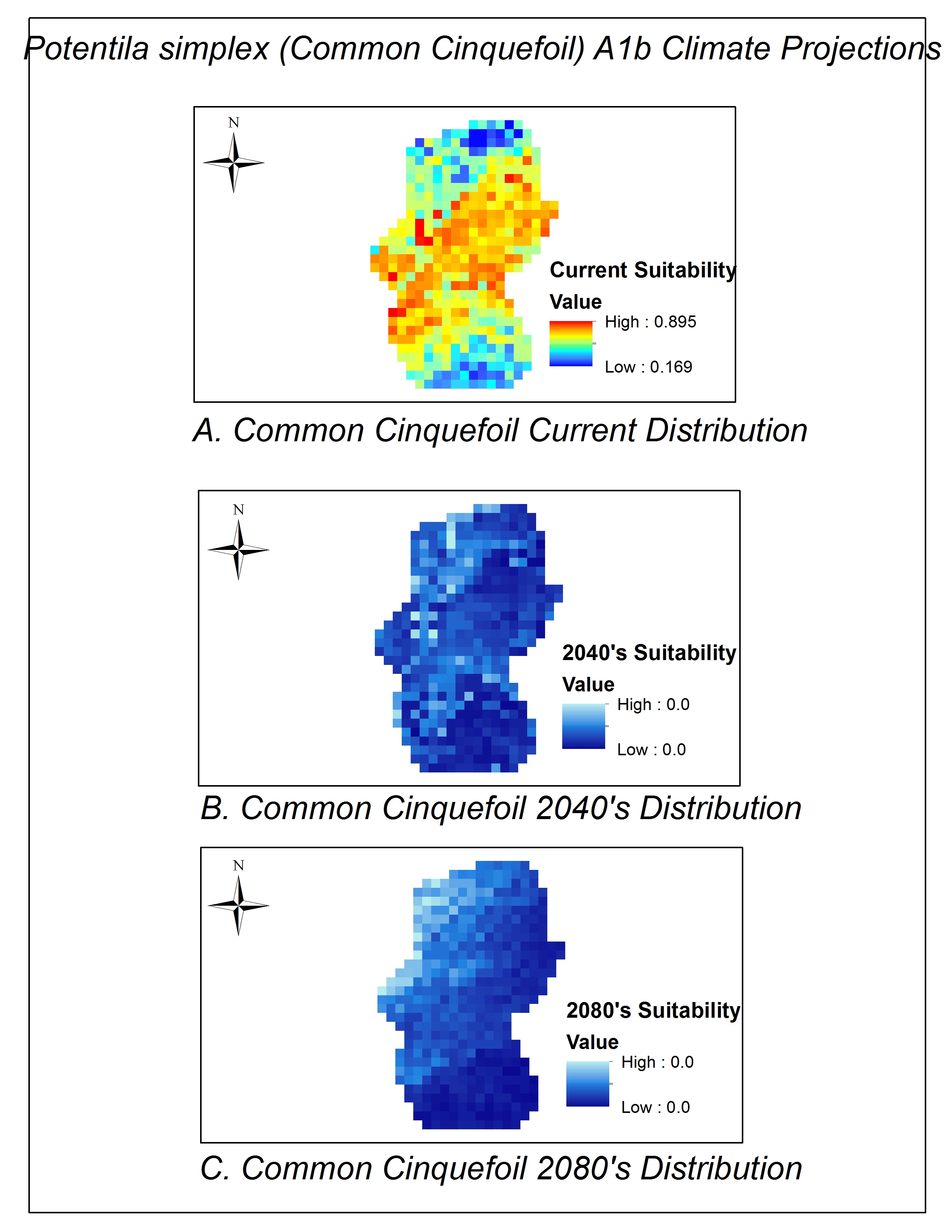


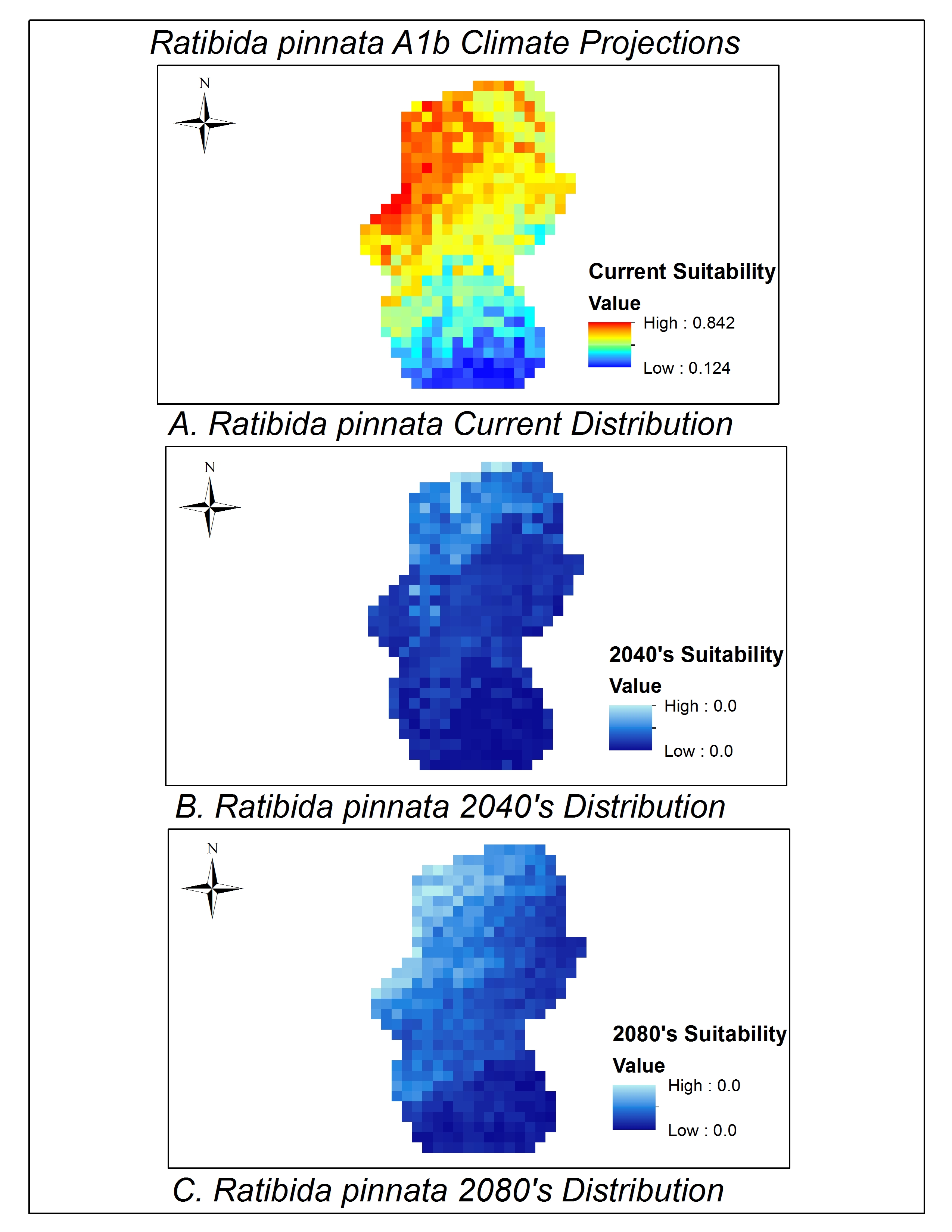


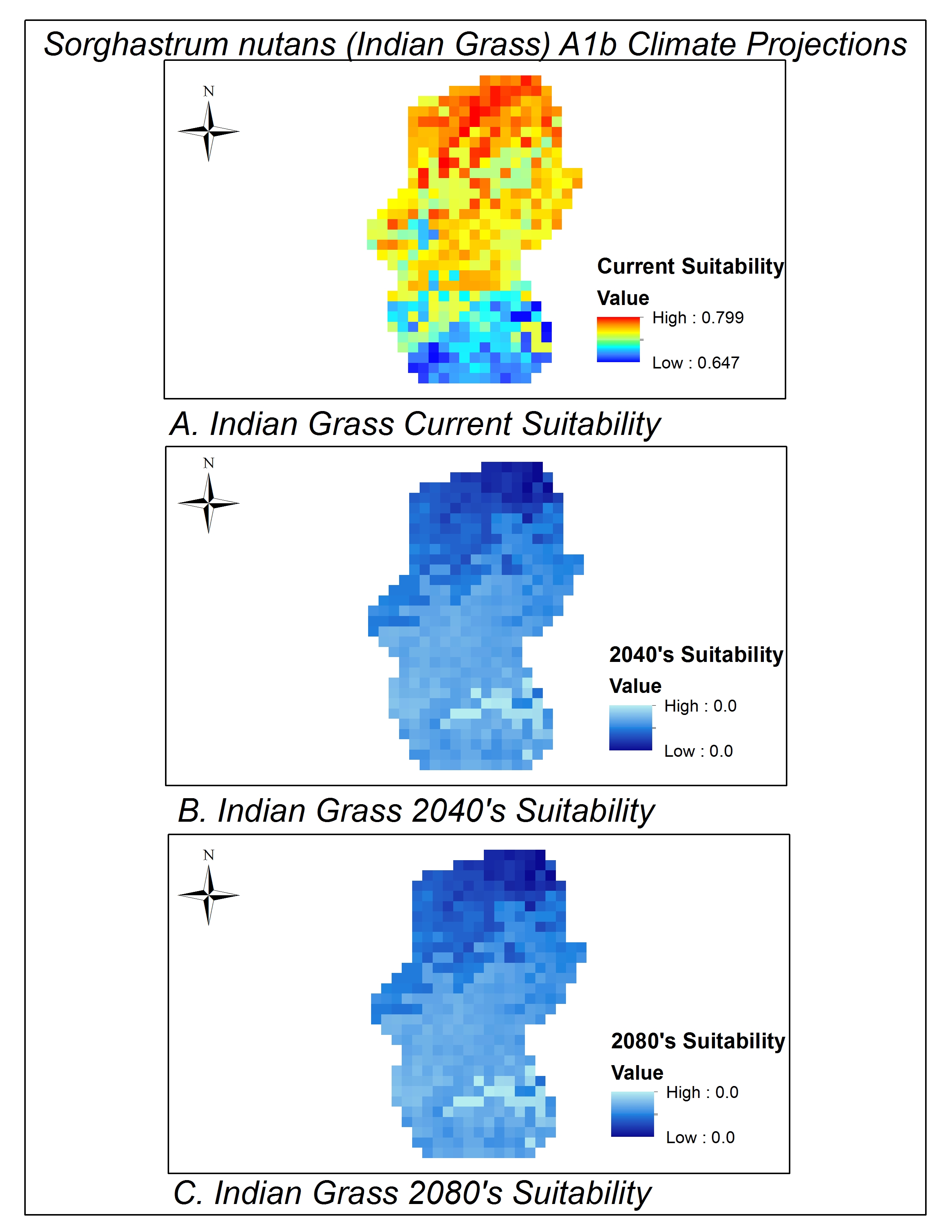


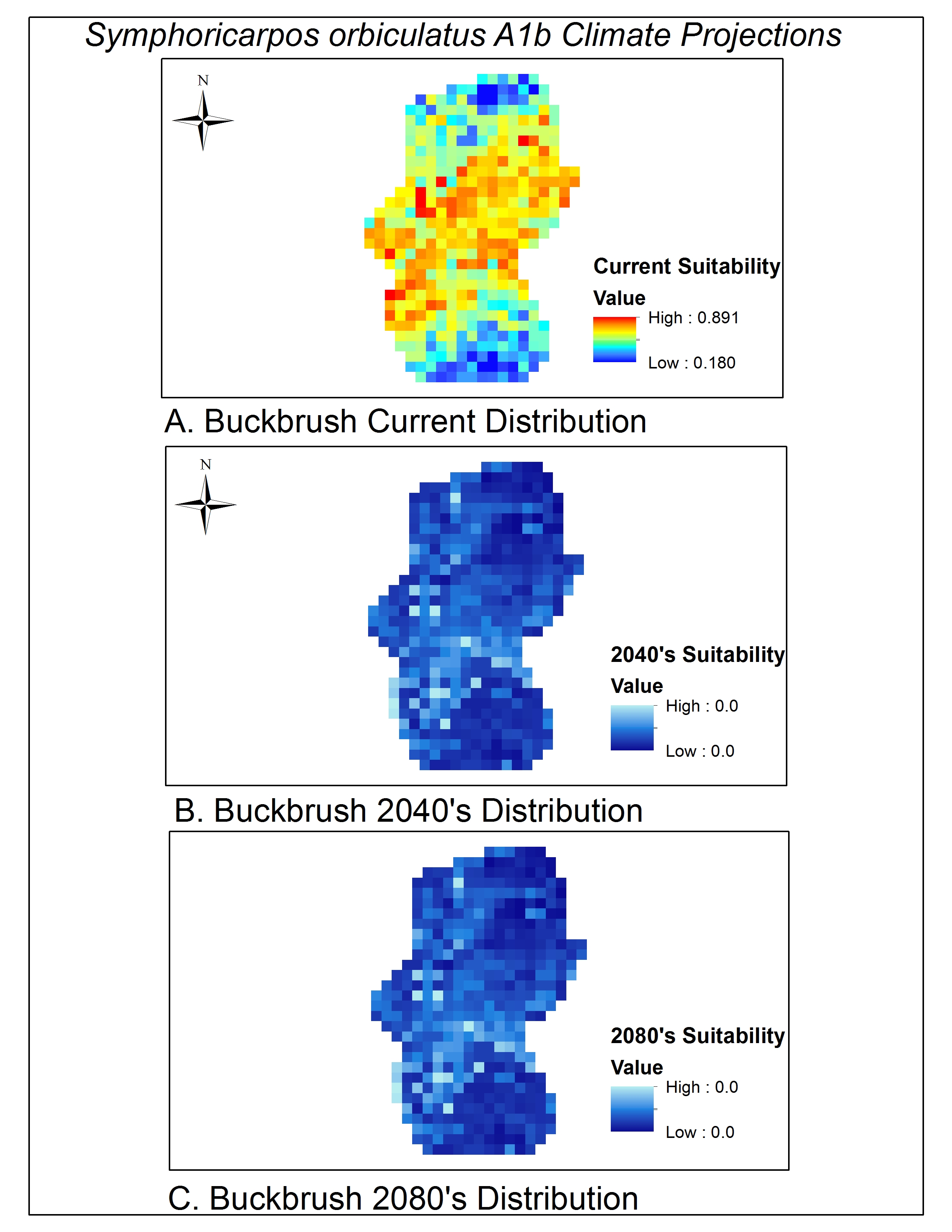


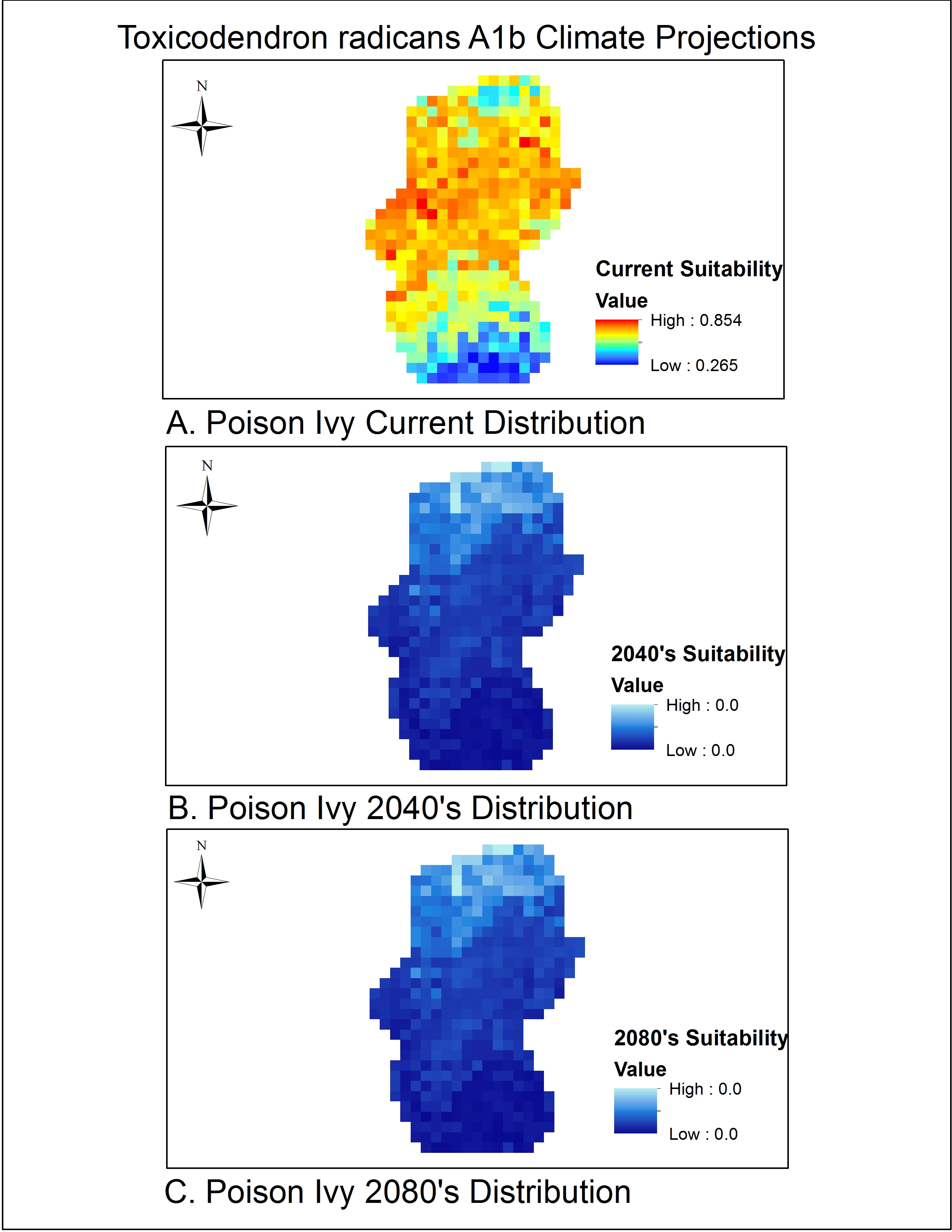

Supplement: Figure S1–S4 — The full set of results for all 33 modeled plant species using the A1B and A2 climate scenarios. [file DataSheet1.DOC]
