# Supplementary material for: Using Regional Climate Projections to Guide Grassland Community Restoration in the Face of Climate Change
Source: Front Plant Sci. 2017 May 9;8:730. doi: 10.3389/fpls.2017.00730 (PMC5422548; doi:10.3389/fpls.2017.00730)
Supplement: Supplementary file 3 [file DataSheet2.DOC]

Supplement B:

A2 Scenario
